# Beyond Selective Serotonin Reuptake Inhibitor (SSRIs): Exploring Hormonal Therapy for Mood Disorders in Perimenopause and Postmenopause

**DOI:** 10.7759/cureus.94752

**Published:** 2025-10-16

**Authors:** Raja Mogallapu, Rebecca Sarich, Ankit Chalia, Michael Ang-Rabanes, Emily Gibson, Venkata Sugnanam, Vijaya Krishna Prasad Vudathaneni

**Affiliations:** 1 Behavioral Health, West Virginia University School of Medicine, Martinsburg, USA; 2 Internal Medicine, Columbus Regional Hospital, Columbus, USA

**Keywords:** estradiol, perimenopause depression hormone replacement therapy (hrt), phq-9, progesterone, vasomotor symptoms

## Abstract

Perimenopause and postmenopause are vulnerable periods for the onset or exacerbation of mood disorders due to fluctuating or persistently low estrogen levels and neurochemical changes. While antidepressants are often first-line treatments, a subset of women may benefit from hormone replacement therapy (HRT), particularly transdermal estradiol. This case report presents two women: one, aged 48, experiencing mood instability during perimenopause with pre-existing depression, and another, aged 55, who developed new-onset depressive symptoms during postmenopause. In both cases, treatment with estradiol alongside careful psychotropic management resulted in marked improvement. These cases highlight the utility of an integrative, hormone-informed approach in managing mood disorders during both perimenopause and postmenopause.

## Introduction

The menopausal transition represents a critical window of vulnerability for mood disturbances in women. Perimenopause, defined as the years immediately preceding menopause marked by cycle irregularity and up to 12 months after the final menstrual period, is characterized by marked fluctuations in ovarian hormone levels, particularly estradiol [1,2]. These fluctuations exert downstream effects on serotonergic, dopaminergic, and noradrenergic neurotransmitter systems, which are central to mood regulation [3,4]. Epidemiological studies demonstrate that women are at increased risk of developing new-onset depression or experiencing recurrence of prior depressive disorders during this phase, even in the absence of psychosocial stressors [5-7]. Postmenopause, in contrast, is defined by chronically low estrogen levels that may perpetuate depressive syndromes in vulnerable individuals [8].

Here, we present two illustrative cases of perimenopausal and postmenopausal depression successfully managed with transdermal estradiol, one in combination with a selective serotonin reuptake inhibitor (SSRI), and the other as a stand-alone therapy. Both cases highlight the importance of distinguishing the reproductive stage in clinical decision-making, the utility of structured assessment tools, and the potential role of multimodal interventions, including hormone replacement therapy (HRT), in optimizing psychiatric outcomes during the menopausal transition.

## Case presentation

Case 1

Ms. A is a 48-year-old female who presented to the outpatient psychiatry clinic with a six-month history of increasingly severe depressive symptoms. This represented a significant transition for her, as she had maintained nearly a decade of remission from major depressive disorder (MDD). This condition was effectively managed through a consistent treatment regimen of escitalopram, administered at a daily dosage of 20 mg.

During her assessment, she reported enduring low mood, emotional lability, fatigue, impaired concentration, and disrupted sleep characterized by mid-night awakenings. Additionally, she exhibited symptoms suggestive of perimenopause, including irregular menstrual cycles, hot flashes, night sweats, and vaginal dryness. When further inquired about her menstrual cycle, the patient indicated that she has been experiencing irregular cycles for the past two years. Notably, her last menstrual period was two weeks before the current psychiatric evaluation. Furthermore, she reported that the symptoms mentioned earlier have consistently aligned with her irregular menstrual cycles and have become more pronounced over the past four weeks. 

Currently denies any symptoms of mania, hypomania, or psychotic symptoms. No suicidal ideations or attempts in her lifetime. Notably, she denied any recent psychosocial stressors or changes in her lifestyle and currently resides with her supportive spouse and two children in a nurturing environment as a housewife.

Ms. A reported no significant medical issues that could contribute to her current state. Her family background revealed mental health concerns, as her mother had been diagnosed with anxiety, while her father dealt with hypertension. Notably, her siblings have not experienced psychiatric hospitalizations, reflecting stable family dynamics. She also denied any substance use in her lifetime.

On the mental status examination, the patient was well-groomed and appropriately dressed, with a depressed mood and mildly labile affect. Her thought processes were logical, and she expressed feelings of helplessness without delusions or suicidal thoughts. She was fully oriented and had generally intact cognition, though with mild attention deficits. Her insight and judgment were fair.

Routine laboratory evaluations, encompassing thyroid function tests and metabolic panels, yielded unremarkable findings. However, reproductive hormone testing indicated characteristics consistent with late perimenopause, including elevated gonadotropins and diminished ovarian steroid production. Ms. A initial Patient Health Questionnaire(PHQ)-9 score was recorded at 16, indicating moderate depression. She was diagnosed with MDD, recurrent, moderate severity, current episode, with mood symptoms likely exacerbated by hormonal changes associated with perimenopause (Tables 1, 2).

**Table 1:** Hormone profile FSH: follicle-stimulating hormone; LH: luteinizing hormone; E2: estradiol

Hormone	Patient value	Reference range (premenopausal)	Reference range (postmenopausal)	Interpretation
FSH	29.6 mIU/mL	4.7-21.5 mIU/mL (follicular phase)	>25.8 mIU/mL	Elevated
LH	15.4 mIU/mL	1.9-12.5 mIU/mL (follicular phase)	7.7-58.5 mIU/mL	Elevated
E2	26 pg/mL	30-400 pg/mL	<30 pg/mL	Low-normal
Progesterone	3 ng/mL	5-20 ng/mL (luteal phase)	<1 ng/mL	Low

**Table 2:** Other lab results TSH: thyroid-stimulating hormone

Test	Patient value	Reference range	Interpretation
TSH	1.0 µIU/mL	0.4-4.0 µIU/mL	Normal
Free thyroxine (free T4)	0.9 ng/dL	0.8-1.8 ng/dL	Normal
Hemoglobin	12.5 g/dL	12-16 g/dL	Normal
Fasting glucose	82 mg/dL	70-99 mg/dL	Normal
Creatinine	0.7 mg/dL	0.6-1.2 mg/dL	Normal
Total cholesterol	195 mg/dL	<200 mg/dL	Normal
LDL cholesterol	80 mg/dL	<130 mg/dL	Normal
HDL cholesterol	50 mg/dL	>50 mg/dL	Normal
Triglycerides	80 mg/dL	<150 mg/dL	Normal
Vitamin D	45 ng/mL	30-100 ng/mL	Normal
Vitamin B12	570 pg/mL	200-900 pg/mL	Normal
Iron studies	Within range	-	Normal

Comprehensive risk screening was performed prior to initiating HRT. The Gail Model for breast cancer risk assessment was applied, incorporating her age, age at menarche, reproductive history, family history of breast cancer, prior breast biopsies, and race/ethnicity. The Gail Model estimated a five-year breast cancer risk of 1.2% (average risk 1.5%) and a lifetime risk of 8.9%(average risk 10.2%).

Cardiovascular risk was assessed using the atherosclerotic cardiovascular disease (ASCVD) Risk Estimator, which accounts for age, sex, race, total cholesterol, HDL cholesterol, blood pressure, smoking status, and diabetes. The ASCVD 10-year cardiovascular risk was 0.8% (low risk category), with normal blood pressure and lipid parameters. Additional labs excluded thyroid dysfunction, anemia, and autoimmune disease.

In light of these findings, she was initiated on treatment with transdermal estradiol at a dosage of 0.025 mg bi-weekly, continuing her regimen of escitalopram. Within two weeks, Ms. A reported a partial improvement in her symptoms, prompting an adjustment of her estradiol dosage to 0.05 mg bi-weekly, along with the introduction of oral micronized progesterone (100 mg nightly) to protect the endometrium and leverage its neuroactive metabolite, allopregnanolone, for anxiolytic and mood-stabilizing effects.

Following the dose adjustments, she experienced significant symptom alleviation after four weeks, resulting in a reduced PHQ-9 score of 11, which indicates mild depressive symptoms. She also noted enhancements in her mood, energy levels, and overall sleep quality with decreased night sweats and hot flashes.

During each follow-up visit, the patient's blood pressure, weight, and symptoms such as leg swelling, breast tenderness, and abnormal uterine bleeding were discussed. No adverse effects were observed, and a gynecologic consultation regarding hormonal therapy confirmed its ongoing safety.

During the three-month follow-up, Ms. A reported continued stability on her regimen of escitalopram 20 mg daily, estradiol 0.05 mg bi-weekly, and progesterone 100 mg nightly. She informed that her night sweats and hot flashes have been in remission for the past month, and her current PHQ-9 score was at 5, indicating minimal depression. After careful review, a shared decision was made to continue the current regimen without any changes.

Case 2

Ms. B is a 55-year-old female who presented to the Department of Psychiatry with persistent depressive symptoms that had become increasingly difficult to manage. Her past medical history is significant for Tourette syndrome, diagnosed at age 12, for which she had previously required psychiatric care during childhood. Over time, her tics gradually diminished in severity, and for the past decade, she has remained stable on aripiprazole 5 mg daily without breakthrough motor or vocal tics.

One year ago, she began experiencing recurrent episodes of depression, which were characterized by a pervasive low mood, irritability, emotional instability, diminished motivation, and reduced energy. These episodes were often exacerbated by vasomotor symptoms, including hot flashes and night sweats, as well as sleep disturbances. These factors further contributed to her fatigue and impaired cognitive function. Each episode lasted for several months, and while there were occasional partial remissions, she never fully regained her previous level of functioning.

When asked about the onset of her symptoms, Ms. B mentioned that she had started to notice subtle changes in her mood since going through menopause at the age of 48. However, these changes had significantly impacted her daily life over the past year.

There is no history of psychiatric illness before menopause, and she denies any trauma history, symptoms suggestive of ADHD, bipolar disorder, or psychosis. She has never engaged in alcohol or illicit drug use, but she was a former smoker. There is no family history of psychiatric disorders, Tourette syndrome, or other major neuropsychiatric illness. Ms. B denied any other medical condition except that she takes an antihistamine for allergies.

Initially, her care was managed by her primary care physician, who prescribed sequential antidepressant trials. She received sertraline (Zoloft), fluoxetine (Prozac), and mirtazapine (Remeron), none of which provided significant clinical benefit despite adequate dosing and trial duration. Venlafaxine XR was then initiated and titrated to 225 mg daily, but this led to severe gastrointestinal side effects, including diarrhea, as well as a marked increase in anxiety. The dose was subsequently reduced to 150 mg daily, which was more tolerable but produced only minimal symptom relief.

As her depression persisted, bupropion was introduced and gradually increased to 450 mg daily. Trials of bupropion in combination with venlafaxine failed to achieve sustained remission. Ultimately, she remained on venlafaxine 150 mg combined with bupropion 450 mg, which offered only modest stabilization of mood and energy. Overall, her antidepressant trials were minimally helpful, with only partial symptomatic control.

As her symptoms worsened and her functional impairment deepened, she was referred to psychiatry for further management. Although the referral had been placed earlier, she initially hesitated to seek psychiatric care, as her only prior experiences had been during childhood for the management of Tourette syndrome. Eventually, the chronicity and severity of her depressive symptoms compelled her to engage with psychiatric services.

She is married, lives with her husband, and has two adult children. She describes a generally supportive home environment, though her persistent mood symptoms have strained relationships at times. Occupationally, she worked in an administrative position for many years, but in recent years, her ability to maintain productivity and focus has been significantly impaired by ongoing fatigue, poor sleep, and diminished motivation.

During the mental status examination, the patient appeared well-groomed and appropriately dressed. Her affect was constricted with mild lability, and her mood was described as depressed and fatigued. Thought processes were logical and goal-directed, with thought content notable for feelings of helplessness but no suicidal ideation, delusions, or hallucinations. Orientation and cognition were intact, and both insight and judgment were assessed as fair.

Routine laboratory work-up, including thyroid and metabolic panels, was within normal limits. Hormonal evaluation confirmed a postmenopausal state with persistently low estradiol and progesterone levels. Additional studies showed normal vitamin D, vitamin B12, and iron indices (Tables 3, 4).

**Table 3:** Hormonal profile FSH: follicle-stimulating hormone; LH: luteinizing hormone; E2: estradiol

Hormone	Patient value	Reference range (premenopausal)	Reference range (postmenopausal)	Interpretation
FSH	41.2 mIU/mL	4.7-21.5 mIU/mL	>25.8 mIU/mL	Elevated
LH	24.6 mIU/mL	1.9-12.5 mIU/mL	7.7-58.5 mIU/mL	Elevated
E2	19 pg/mL	30-400 pg/mL	<30 pg/mL	Low
Progesterone	1.5 ng/mL	5-20 ng/mL (luteal phase)	<1 ng/mL	Low-normal

**Table 4:** Lab results TSH: thyroid-stimulating hormone

Test	Patient value	Reference range	Interpretation
TSH	2.1 µIU/mL	0.4-4.0 µIU/mL	Normal
Free T4	1.1 ng/dL	0.8-1.8 ng/dL	Normal
Fasting glucose	92 mg/dL	70-100 mg/dL	Normal
Total cholesterol	185 mg/dL	<200 mg/dL	Normal
LDL cholesterol	95 mg/dL	<130 mg/dL	Normal
HDL cholesterol	58 mg/dL	>50 mg/dL	Normal
Triglycerides	110 mg/dL	<150 mg/dL	Normal
Serum creatinine	0.8 mg/dL	0.6-1.1 mg/dL	Normal
AST/ALT	20/22 U/L	<40 U/L	Normal
Vitamin D	52 ng/mL	30-100 ng/mL	Normal
Vitamin B12	420 pg/mL	200-900 pg/mL	Normal
Iron studies	Within range	-	Normal

According to DSM-5 criteria, her depressive symptoms were consistent with MDD due to another medical condition (postmenopausal hypoestrogenism), characterized by depressed mood, diminished interest or pleasure, fatigue, sleep disturbance, irritability, and impaired occupational functioning, with symptoms emerging after menopause and not explained by other psychiatric disorders.

The Gail Model estimated a five-year breast cancer risk of 1.3% (average risk 1.5%) and a lifetime risk of 9.4%(average risk 10.2%), and the ASCVD Risk Estimator revealed a 1.5% (low risk category) for cardiovascular disease. Laboratory studies excluded thyroid dysfunction, anemia, and autoimmune disease.

Given inadequate response to multiple antidepressant regimens and confirmed hypoestrogenism, the patient expressed a preference not to continue antidepressants, as she found them minimally helpful. After discussion of risks and benefits, a shared decision-making approach was employed, and venlafaxine and bupropion were gradually tapered off over a three-week period.

She was initiated on transdermal estradiol 0.025 mg/bi-weekly with oral micronized progesterone 200 mg nightly for endometrial protection. At baseline, the patient's PHQ-9 score was 18, consistent with moderately severe depression. Following six weeks of hormone therapy, her score improved to 6, reflecting mild depressive symptoms. Transdermal estradiol patch at a dose of 0.025 mg was titrated to 0.1 mg bi-weekly with continuation of oral micronized progesterone (200 mg nightly), alongside the gradual taper and discontinuation of venlafaxine and bupropion over six weeks per shared decision-making.

Her PHQ-9 score further improved to 3 at a three-month follow-up, indicating near-complete remission. The Menopause Rating Scale (MRS), which has a maximum possible score of 44, was administered both before and after treatment. The patient’s total score improved significantly, decreasing from 30 before treatment to 8 after treatment. There were notable reductions in all three domains: psychological (from 12 to 3), somatic (from 10 to 3), and urogenital (from 8 to 2). These results reflect substantial improvement in symptoms (Tables 5, 6).

**Table 5:** MRS comparison MRS: Menopause Rating Scale

Domain	Key symptoms	Baseline score	Post-treatment score	Change
Somatic	Hot flashes, night sweats, heart discomfort, and sleep problems	10	3	↓ 7
Psychological	Depressed mood, irritability, anxiety, and fatigue	12	3	↓ 9
Urogenital	Vaginal dryness, bladder issues, and sexual dysfunction	8	2	↓ 6
Total MRS Score	44	30	8	↓ 22

**Table 6:** PHQ-9 score comparison and antidepressant status

Metric	Ms. A (pre-existing depression)	Ms. B (new-onset depression)
Baseline PHQ-9	16 (moderate)	18 (moderately severe)
After estradiol initiation	11 (mild)	6 (mild)
At 3-month follow-up	5 (minimal)	3 (minimal)
Antidepressant status	Continued lexapro 20 mg	Discontinued venlafaxine, wellbutrin

At each follow-up visit, the patient’s blood pressure, weight, and review of systems for leg swelling, breast tenderness, and abnormal uterine bleeding were conducted. No adverse effects were noted, and gynecologic follow-up confirmed the continued safety of hormone therapy. Clinically, she reported a marked improvement in sleep quality, resolution of hot flashes, reduced irritability, enhanced emotional stability, and increased motivation and productivity at work. 

She was maintained on her established pharmacotherapeutic regimen, comprising aripiprazole 5 mg once daily for the management of Tourette syndrome, transdermal estradiol (0.1 mg) administered biweekly, and oral micronized progesterone 200 mg nightly. No modifications were made to her ongoing treatment plan.

## Discussion

These two cases illustrate the complex interplay between hormonal fluctuations during the perimenopausal and postmenopausal stages and mood disturbances. Ms. A, with a history of major depressive disorder, experienced a relapse during the perimenopausal transition, while Ms. B began to experience new-onset depressive symptoms after entering menopause. In both patients, laboratory and clinical evidence confirmed their reproductive stage, and both underwent gynecological evaluation in the context of HRT. Mood symptoms during perimenopause and postmenopause are common and may not respond adequately to antidepressants alone. The integration of transdermal estradiol into their treatment plans led to substantial mood improvement, aligning with existing literature on estrogen’s mood-modulating role [1,2].

Perimenopause is clinically defined as the period immediately preceding menopause, characterized by menstrual cycle irregularities, and extending up to 12 months after the final menstrual period [2,3]. This transition can last several years, during which fluctuating estrogen levels frequently contribute to vasomotor, cognitive, and affective symptoms. By contrast, postmenopause refers to the time beginning 12 months after the final menstrual period, during which estrogen levels stabilize at chronically low levels [4]. Both stages can be associated with mood disturbances, although the mechanisms differ: perimenopause is marked by fluctuating hormonal activity, while postmenopause reflects sustained estrogen deficiency.

Estrogen plays a significant role in modulating serotonergic, dopaminergic, and noradrenergic systems-neurochemical pathways commonly targeted in the pharmacological treatment of depression [5,6]. During perimenopause, declining and fluctuating estrogen levels disrupt these systems, increasing the risk of mood instability even in individuals with no prior psychiatric history [7,8]. In postmenopause, the persistent estrogen deficiency can similarly contribute to depressive syndromes, especially in vulnerable women [9,10].

Transdermal estradiol is particularly beneficial due to its stable serum concentrations and lower risk profile for venous thromboembolism compared to oral formulations [11,12]. Several randomized controlled trials and observational studies have shown that estradiol exerts antidepressant effects in both perimenopausal and postmenopausal women, either in combination with or as an alternative to SSRIs or serotonin and norepinephrine reuptake inhibitors (SNRIs) [13,14].

In Ms. A’s case, augmentation of Lexapro with estradiol resulted in significant symptom relief. In Ms. B’s case, estradiol allowed for eventual tapering off of antidepressants altogether, a notable outcome given her poor tolerance to multiple psychotropics. These observations support the idea that, for some women, estrogen-based therapy may address the underlying hormonal mechanisms contributing to affective symptoms [5,14].

Progesterone also contributes to mood regulation. Through its neuroactive metabolite allopregnanolone, progesterone modulates the GABA-A receptor, exerting anxiolytic, sedative, and mood-stabilizing effects [15]. Micronized progesterone has been associated with improved sleep quality and reductions in anxiety-related symptoms, particularly when administered at bedtime. When combined with estradiol, progesterone may provide synergistic benefits for mood regulation and vasomotor symptom relief [15]. Importantly, bioidentical micronized progesterone is generally preferred over synthetic progestins such as medroxyprogesterone acetate, given its more favorable neuropsychiatric profile and reduced risk of mood destabilization [15]. Allopregnanolone, the primary active metabolite, underlies the development of brexanolone and zuranolone, FDA-approved and investigational treatments for major depressive disorders [16].

Both patients were comprehensively screened for HRT safety using the Gail Model for breast cancer risk assessment and the ASCVD Risk Estimator [17-20]. The Gail Model incorporates factors such as age, age at menarche, age at first live birth, family history of breast cancer, prior breast biopsies, and race/ethnicity to provide a five-year and lifetime risk estimate [17]. The ASCVD Risk Estimator considers age, sex, race, total cholesterol, HDL cholesterol, blood pressure, diabetes, and smoking history to estimate the 10-year and lifetime risk of atherosclerotic events [18]. Both patients demonstrated low-risk profiles, allowing initiation of HRT without contraindications.

Other potential medical contributors to mood disturbance, such as thyroid dysfunction, anemia, autoimmune conditions, and substance use, were ruled out. HRT contraindications, including active or prior estrogen-sensitive cancers, unexplained vaginal bleeding, active or prior thromboembolic events, severe liver disease, and uncontrolled hypertension, were carefully considered in both cases [19,20]. Long-term planning for HRT requires not only risk assessment but also structured monitoring, including periodic pelvic exams, mammography, and ongoing thromboembolic risk evaluation.

The off-label use of estradiol for mood symptoms by psychiatrists presents notable limitations. These include limited gynecologic expertise in managing estrogen-sensitive conditions, gaps in monitoring capacity, and regulatory concerns around prescribing HRT for non-gynecologic indications. Both patients were extensively counseled about these issues, underwent gynecological evaluation prior to treatment initiation, and were referred for ongoing gynecologic follow-up. Importantly, the psychiatric treatment team remained in close coordination with the gynecologists, ensuring aligned adjustments in care and continuity of monitoring. This interdisciplinary collaboration underscores the importance of integrating psychiatric and gynecologic care in managing mood disorders during midlife hormonal transitions.

Both cases highlight the utility of standardized assessment tools in evaluating treatment response. The PHQ-9 allowed systematic tracking of depressive symptoms over time and was selected for its feasibility and practicality in routine clinical practice. In the case of Ms. A, the PHQ-9 was administered to evaluate depressive symptoms. However, no standardized scale specific to perimenopausal depression was utilized, as there are presently no validated instruments designed exclusively to assess depressive symptomatology within the perimenopausal context.

Recent literature has drawn attention to the Meno-D scale, a menopause-specific depression measure currently undergoing validation, developed to identify and quantify mood disturbances related to hormonal fluctuations more accurately. The Meno-D has demonstrated promising reliability and construct validity in preliminary studies and will be considered for inclusion in future assessments for a more comprehensive evaluation of mood symptoms during perimenopause [21].

In Ms. B’s case, the MRS was additionally used, which provided a structured assessment of somatic, psychological, and urogenital symptoms associated with menopause [22]. The substantial reduction in Ms. B’s MRS score (30 to 8) highlighted not only mood improvement but also alleviation of broader menopausal symptomatology, underscoring the importance of multidimensional outcome measures in this population.

## Conclusions

Mood symptoms during perimenopause and postmenopause are common and may not respond adequately to antidepressants alone. Incorporating transdermal estradiol into treatment plans can improve outcomes and, in some cases, reduce the need for antidepressants. These cases emphasize the importance of defining the reproductive stage, perimenopause versus postmenopause, as both fluctuating and chronically low estrogen levels can contribute to depressive syndromes. Hormonal therapy should be considered as part of a comprehensive treatment approach that includes thorough screening, patient education, and interdisciplinary collaboration with gynecologists. Both patients were referred to gynecology, followed up closely, and their psychiatric treatment was coordinated with gynecologic care to ensure alignment and safety.

Detailed risk assessment through tools such as the Gail Model and ASCVD Risk Estimator ensures safety, while long-term monitoring supports sustained treatment benefit. Standardized assessment tools, including the PHQ-9 and MRS, are valuable for tracking treatment response and symptom changes. However, there is currently no universally accepted scale specific to the perimenopausal transition, which limits precision in both clinical practice and research. Future studies should prioritize the development and validation of perimenopause-specific instruments to better capture the dynamic interplay between hormonal fluctuations and psychiatric outcomes. Prospective, controlled studies are needed to clarify the biological mechanisms linking hormonal changes to mood disturbances and to further delineate the efficacy of both HRT and adjunctive treatments in perimenopausal and postmenopausal depression. Distinguishing correlation from causation will ultimately guide safer, more effective, and personalized treatment strategies for women undergoing these critical life transitions. Healthcare providers should maintain a high level of vigilance when working with this age group, as there may be a new onset of mood symptoms, subtle mood fluctuations, or exacerbation of pre-existing mood disorders.
